# Metabolic factors are not the direct mediators of the association between type 2 diabetes and osteoporosis

**DOI:** 10.3389/fendo.2024.1404747

**Published:** 2024-07-25

**Authors:** Qifan Yang, Xinyu Wang, Yanwei Liu, Jing Liu, Dong Zhu

**Affiliations:** ^1^ Department of Orthopedics, The First Hospital of Jilin University, Changchun, China; ^2^ Department of Gynecology and Obstetrics, Jinan Maternity and Child Care Hospital Affiliated to Shandong First Medical University, Jinan, China

**Keywords:** T2DM, type 2 diabetes mellitus, osteoporosis, OS, Mendelian randomization

## Abstract

**Objective:**

The causal relationship between type 2 diabetes mellitus (T2DM) and osteoporosis (OS) remains unclear. This study aims to investigate the causal relationship and explore the potential metabolic mechanism and its mediating role.

**Methods:**

We conducted a comprehensive study, gathering data on 490,089 T2DM patients from the genome-wide association study (GWAS) database and selecting OS data from FinnGen and MRC-IEU sources, including 212,778 and 463,010 patients, respectively, for causal analysis. Simultaneously, we explored the potential roles of three obesity traits and 30 metabolic and inflammation-related mediating variables in the causal relationship.

**Results:**

There is a strong causal relationship between T2DM and OS. The data from our two different database sources appeared in the same direction, but after correcting for body mass index (BMI), waist circumference (WC), and waist-to-hip ratio (WHR), the direction became the same. T2DM may increase the risk of OS [odds ratio (*OR*) > 1.5, *p* < 0.001]. Steiger’s test results show that there is no reverse causality. No risk factors related to glycolipid metabolism, amino acid metabolism, and inflammation were found to mediate the causal relationship.

**Conclusion:**

This study’s findings indicate a robust causal relationship between T2DM and OS, influenced by relevant factors such as BMI. Our results shed light on the pathogenesis of OS and underscore the importance for clinicians to treat metabolic disorders to prevent osteoporosis.

## Introduction

Abnormal glucose metabolism, a condition characterized by the body’s poor absorption, transport, and glucose metabolism, is a significant health concern. This includes impaired fasting glucose, abnormal glucose tolerance, and diabetes. The prevalence of diabetes will rise dramatically, reaching 782.1 million individuals (approximately 12.2% of the adult population) by 2045 (1). This alarming trend underscores the importance of understanding the relationship between abnormal glucose metabolism and other health complications, such as bone degeneration. Notably, approximately 35% of patients with type 2 diabetes mellitus (T2DM) experience abnormal glucose metabolism and bone degeneration, leading to osteoporosis (OS), a chronic bone complication that poses a serious threat to human health (2). Research has demonstrated that T2DM adversely affects bone metabolism, reducing bone mass and increasing bone fragility and fracture risk.

Furthermore, the impact of T2DM on bone health is not straightforward. It affects not only bone geometric characteristics (3–6), bone composition, microstructure, and mechanical properties but also intricate underlying mechanisms (7, 8). For instance, T2DM can lead to bone loss by damaging the primary cilia of osteoblasts. This process involves the generation of excessive reactive oxygen species (ROS), which further disrupts osteoblasts’ mitochondrial metabolism and primary cilia structure. Currently, there are numerous basic studies on T2DM and OS. However, the potential role of lipid metabolism, amino acid metabolism, and inflammatory factors in these mechanisms should be fully explored.

As of now, the globalization trend of OS has accelerated. At present, studies have proved that different fatty acids can affect osteoclasts. Omega-3 polyunsaturated fatty acids and omega-7 fatty acids can reduce the number of osteoclasts, inhibit the downstream signal activation of RANKL, and reduce bone loss (9). Excessive inflammatory cytokines lead to increased bone absorption and decreased bone mass. ROS scavenging hydrogel can significantly inhibit inflammatory factors and reduce ROS in bone tissue of patients with OS complicated with hips; hydrogen peroxide (H_2_O_2_), hydroxyl radical (OH^−^), and superoxide anion (O_2_^−^) increase the expression of osteoblast-related factors (10, 11). Basic research on T2DM and OS has been abundant, but we still need to further explore the genetic relationship between various metabolic factors and OS.

Given the lack of consideration of confounding factors and lack of proof of causality, this study adopted Mendelian randomization (MR) analysis to analyze the causality between T2DM and OS. MR represented relevant traits by instrumental variable (IV) to explore the inherent causality of heredity. Because life follows random allocation of chromosomes at conception, MR analyses resemble clinical randomized trials and are less susceptible to confounding and reverse causality. We analyze different factors that may affect T2DM and OS through multivariate analysis [multivariable MR (MVMR)] and mediation analysis. As far as we know, this MR study, for the first time, included the analysis of glucose metabolism, lipid metabolism, amino acid metabolism, and inflammatory factors in the causal relationship between T2DM and OS. We hope this article can provide relevant diagnosis and treatment ideas for clinicians and researchers to study the pathogenesis and treatment of T2DM and OS.

## Materials and methods

### Study design

**Figure 1:**
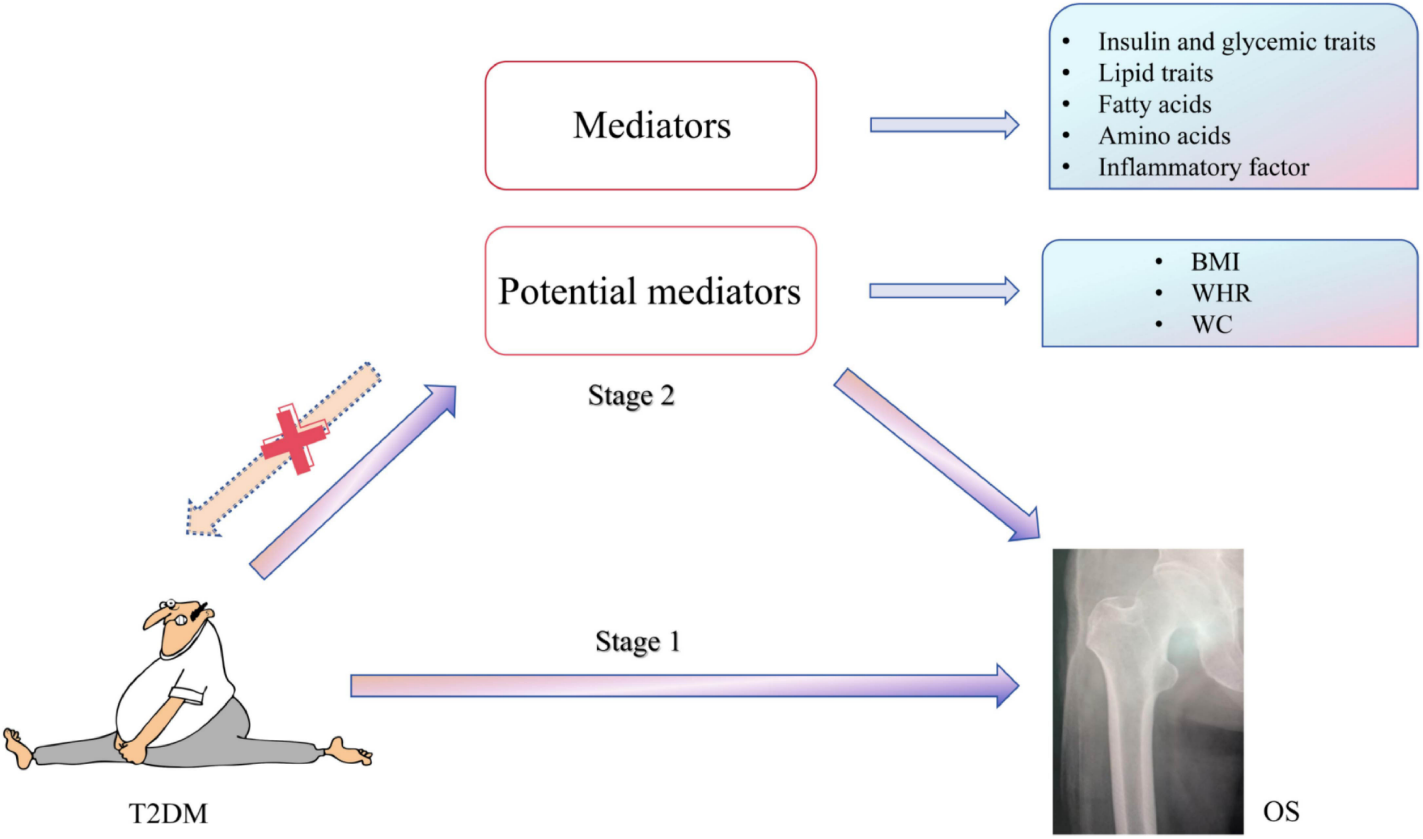
MR study design. Stage I: MR analysis of T2DM and OS samples was performed. Stage 2: to explore the potential role of obesity indicators and mediating variables in causality. MR, Mendelian randomization; T2DM, type 2 diabetes mellitus; OS, osteoporosis.

During the study, we always followed the STROBE-MR Guidelines (**Supplementary Table 1**) (12) and insisted on three basic assumptions of MR: first, IVs are strongly correlating with exposure factors (T2DM). Second, IVs are not associated with confounding factors between T2DM and OS. Third, IVs affect OS only through T2DM and not through any direct or indirect route. All original studies cited in this study have received ethical approval and informed consent. This study consists of three stages. In the first phase, we conducted a univariate MR study of T2DM and OS using pooled data from a genome-wide association study (GWAS) to assess the causal relationship between the two. In the second stage, we used MVMR to analyze obesity indicators: waist-to-hip ratio (WHR), body mass index (BMI), and waist circumference (WC). We used mediating MR to evaluate the effects of glucose metabolism, lipid metabolism, amino acids, and inflammatory factors. In the third stage, we verified the OS data from “UKB-B-17796” (**Figure 1**), indicating the MR hypothesis followed the study design.

### Data sources for and selection of genetic instruments

**Table 1** summarizes the database information involved in this study and sets the selection criteria for single-nucleotide polymorphisms (SNPs) as *p* < 5 × 10^−8^, R^2^ = 0.001, and window = 10,000 kb based on the significance level of the whole genome (13). In order to ensure that the selected IVs have sufficient strength, we used F-value >10 as the inclusion criterion (14) and eventually used the included SNPs for subsequent MR analysis.

### Type 2 diabetes

IVs associated with T2DM were obtained from the European Bioinformatics Institute (EBI) (15), PMID: 34594039, GWAS ID: ebi-a-GCST90018926, including 38,841 cases and 451,248 controls, adjusted for sex. The diagnostic criteria adopted for T2DM were 1) glycosylated hemoglobin (HbA1c) ≥6.5% and 2) fasting glucose level >125 mg/dL, and **Supplementary Table 3** shows the specific SNP information.

### Adiposity traits

T2DM patients are often associated with obesity, but the inclusion database of exposure factors did not adjust BMI. In this study, adult BMI, adult WC, and WHR were included for multivariate analysis. BMI (PMID: 34017140) (16) was a study of 407,746 individuals of white British ancestry in the UK Biobank database, and 471,762 genotyping SNPs were retained through screening. WC (PMID: 35399580) (17) was derived from a study of 70,409 Hispanics/Latinos; WHR (PMID: 29892013) (18) was derived from 10,759 independent GWAS sites.

### Candidate mediators

T2DM is closely related to glucose metabolism, lipid metabolism, fatty acid metabolism, amino acid metabolism, and inflammatory factors (19–24). Databases with baseline characteristics similar to exposure and outcomes were collected as data sources for candidate moderators (**Table 1**). In the course of onset, T2DM patients always have metabolic disorders (16, 25, 26), so metabolic factors were focused on, which include four indications of glucose metabolism—fasting insulin (FI), fasting blood glucose (FG), 2-H glucose, and HbA1c—with the maximum sample sizes for each of the first three traits ranging from 85,916 (2-H glucose) to 281,416 (FG), and participants of European ancestry dominated the sample sizes for all traits (27). HbA1c data were derived from a 1,000,000 population sample of white British or UK Biobank’s complete European ancestry set, stratified among first-, second-, and third-degree relatives of participants. In terms of lipid metabolism factors, apolipoprotein A-I (Apo A-I), apolipoprotein B (APO-B), high-density lipoprotein cholesterol (HDL-C), low-density lipoprotein cholesterol (LDL-C), and triglycerides (TG) were included, among which the Apo A-I and APO-B data came from 393,193 and 439,214 samples in the UKB database, containing 440 and 255 genetic variants, respectively (25). HDL-C was also derived from the above HbA1c database, and LDL-C data were used from the UK Biobank (n = 431,167), the Global Lipids Genetics Consortium (n = 188,577), and the Consortium for Diabetes Genetics Replication and Meta-Analysis (n = 188,577) (26). HDL-C was attained from the above HbA1c database, and LDL-C data were used from the UK Biobank (n = 431,167), the Global Lipids Genetics Consortium (n = 188,577), and the Consortium for Diabetes Genetics Replication and Meta-Analysis (n = 188,577) (28), and TNF-α and IL-17 data were derived from GWAS of 8,293 Finnish individuals (29). The European ancestry provides IL-1β information on approximately 2,994 protein analyses in 3,301 individuals. The rest include seven fatty acids [saturated fatty acids (SFA), monounsaturated fat acids (MUFA), polyunsaturated fat acids (PUFA), omega-3 fatty acids, omega-6 fatty acids, docosahexaenoic acid (DHA), and linoleic acid] and nine amino acids (isoleucine, leucine, valine, phenylalanine, tyrosine, alanine, glutamine, glycine, and histidine).

**Table 1:** MR results for the causal effect of type 2 diabetes on osteoporosis.

Outcome	Methods	No. of SNPs	F statistic	*OR*	95% *CI*—low	95% *CI*—up	*p-*Values
Osteoporosis	MR-Egger	235	89	0.94	0.83	1.07	0.363
	Weighted median	235		0.87	0.79	0.96	0.005
	IVW	235		0.890	0.84	0.94	< 0.001
Osteoporosis (UKB-B-17796)	MR-Egger	180	89	1.09	1.01	1.17	0.032
	Weighted median	180		1.06	1.00	1.11	0.035
	IVW	180		1.05	1.02	1.09	0.003

MR, Mendelian randomization; SNPs, single-nucleotide polymorphisms; IVW, inverse variance weighting.

### OS

The Finnish database included 3,203 patients and 209,575 controls, with 16,380,452 SNPs, which we selected for OS. The MRC-IEU database provided the validation OS database, which included 1,976 patients and 461,034 controls with 9,851,867 SNPs.

### Statistical analyses

#### Univariable and multivariable MR analyses

This study used MR-Egger, weighted median, inverse variance weighting (IVW), simple model, and weighted model function to analyze the two-sample MR. The IVW method used meta-analysis to process the Wald ratio estimate of SNP into causality value, which was selected as the most accurate method (13). Statistical values of Cochran’s Q test evaluate heterogeneity. According to the MR-Egger regression model, judgments were made according to intercept and *p*-values to conduct a horizontal pleiotropic analysis (30, 31). “MRlap” was used to measure the sample overlap rate between exposure and outcome, and the results were respectively 0.7% and 0.4%, with no bias. To eliminate other SNP interference, SNPs associated with other traits of T2DM were excluded using the LDtrait tool (https://ldlink.nih.gov/?tab=ldtrait), and Steiger’s test was performed (32).

In the MVMR analysis, according to the results of multivariate inverse variance weighting (MV-IVW), it was determined whether obesity traits partially mediated the causal relationship between T2DM and OS. Multivariate Mendelian randomized Egger (MVMR-Egger) and MV-IVW methods were used for sensitivity analysis.

#### Effect of T2DM on OS

In this study, we used two-sample MR to analyze the causal association between T2DM and OS and odds ratio (*OR*) and 95% confidence interval (*CI*) to determine risk events. The MR-PRESSO method detects and removes abnormal SNPs to exclude their influence on horizontal pleiotropy (33). T2DM patients are often associated with obesity, and both have a strong genetic susceptibility to OS. Therefore, in this study, obesity indicators BMI, WC, and WHR were considered potential confounders and analyzed using the MVMR method. For the results of the two-sample MR and MVMR, we verified the OS database with GWAS ID: “UKB-B-17796”.

### Mediation analysis

**Figure 2:**
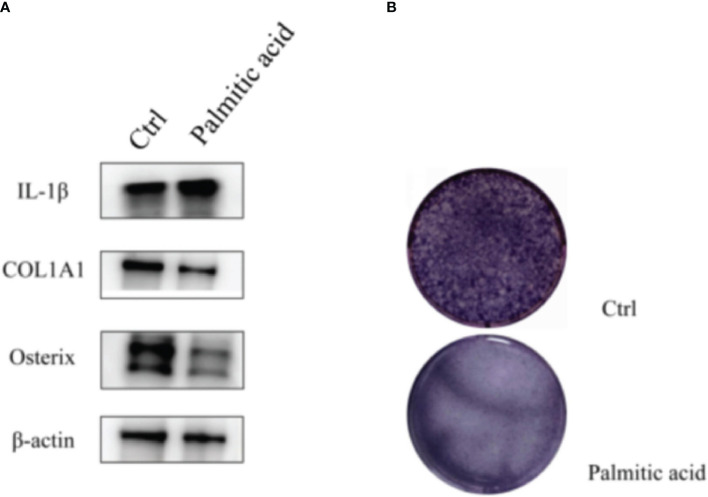
T2DM can induce changes in inflammatory factors and osteogenic proteins. **(A)** Western blotting: palmitic acid decreases the expression of osteogenic proteins and increases the expression of inflammatory factors. **(B)** Alkaline phosphatase staining: osteoblasts were treated with palmitic acid, and their osteogenic differentiation ability decreased. T2DM, type 2 diabetes mellitus.

In the preliminary experiments of this study, it was found that the disorder of glucose metabolism and lipid metabolism would reduce osteogenic proteins and increase the expression of inflammatory factors (**Figure 2**). Therefore, 30 candidate moderating variables associated with T2DM and OS were searched. Two-sample MR analysis (β1) was performed on T2DM and mediating variables to screen out 17 causal mediating factors, and then heterogeneity and horizontal pleiotropy analysis were performed. The existing level of pleiotropic intermediary factors on http://www.phenoscanner.medschl.cam.ac.uk/ eliminate the SNPs associated with other exposures; MVMR further analyzed the 17 mediating factors and T2DM to quantify the possible mediating effect between T2DM and OS (β2). All MR analyses were performed on R software (R version 4.2.2), involving R packages including “TwoSampleMR”, “MRPRESSO”, “MRlap”, and “MendelianRandomization”.

#### Western blotting

Western blotting assay extracts proteins from cell samples by radioimmunoprecipitation assay (RIPA) lysis buffers and protease and phosphatase inhibitors. The protein sample was dissolved on ice for 20 min, mixed with 5× sodium dodecyl sulfate–polyacrylamide gel electrophoresis (SDS-PAGE) sample loading buffer (WB3002; NCM Biotech, Newport, RI, USA), and heated at 100°C for 5 min. After cooling, denatured proteins were isolated in SDS-PAGE gel (PG112; Epizyme, Cambridge, MA, USA). They were transferred to polyvinylidene fluoride (PVDF) membrane (Millipore Corp., Billerica, MA, USA), sealed with a rapid sealing solution (PS108P; Epizyme), and combine with Collagen I (ab268843; Abcam, Cambridge, UK), Osterix (ab209484; Abcam), IL-1β (31202S; CST, Danvers, MA, USA), and β-actin (AF0003; Beyotime, Shanghai, China). They were incubated overnight at 4°C with gentle shaking. On the second day, the membrane and horseradish peroxidase (HRP)-labeled secondary antibodies were incubated at room temperature for 1 hour. Color development was performed using a supersensitive ECL chemiluminescence kit (P10300; NCM Biotech), and imaging was performed on a chemiluminescence gel imaging system (Fusion Solo S; Vilber, Collégien, France). The gray values of the strips were analyzed using ImageJ software 1.53a (Wayne Rasband, National Institutes of Health, USA).

#### Alkaline phosphatase staining

Alkaline phosphatase staining was conducted using a BCIP/NBT alkaline phosphatase chromogenic kit (Beyotime, China). The cells were spread into six-well plates at 15w/well and cultured with ordinary medium or medium containing 0.3 mM palmitic acid for 7 days on the second day; the medium was changed every 3 days. The cells were flushed with phosphate-buffered saline (PBS) during staining and then fixed with neutral buffered formalin (10%) for 15 min. The cells were then flushed, and a liquid substrate of BCIP/NBT was added to each well. Finally, after the cell color turned blue/purple, the cells were washed with ddH_2_O and photographed.

## Results

### Impact of T2DM on OS

**Figure 3:**
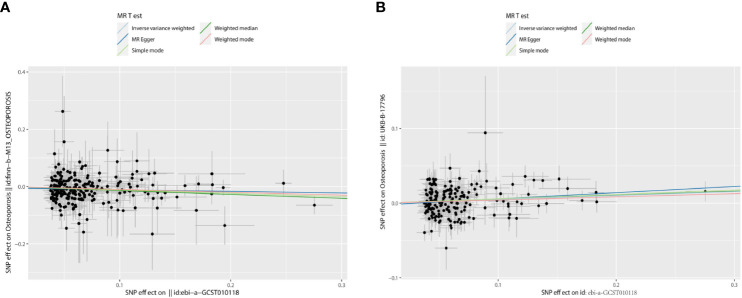
The scatter plots: causal relationship between T2DM and OS. **(A)** Osteoporosis. **(B)** Osteoporosis (U KB-B-17796). T2DM, type 2 diabetes mellitus; OS, osteoporosis.

**Figure 4:**
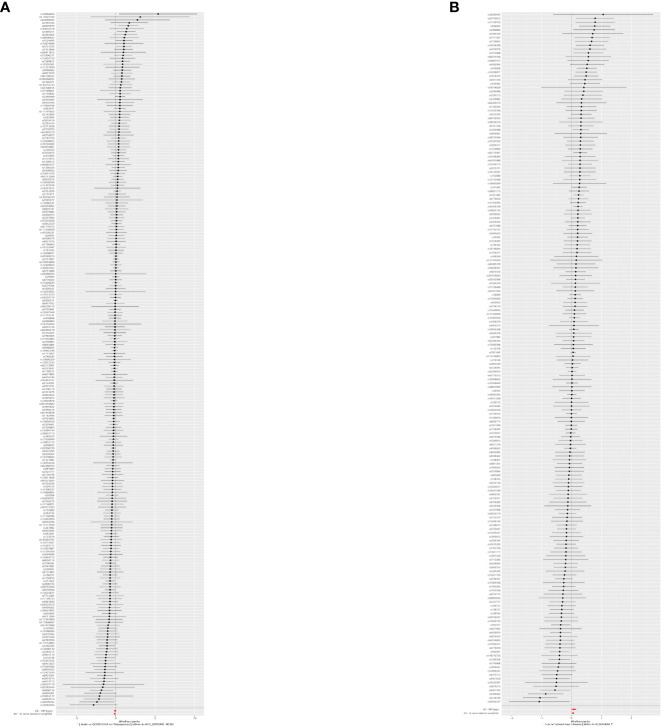
The forest plot: causal relationship between T2DM and OS. **(A)** Osteoporosis (finn-b-M13_OSTEOPOROSIS). **(B)** Osteoporosis (UKB-B-1 7796). T2DM, type 2 diabetes mellitus; OS, osteoporosis.

**Figure 5:**
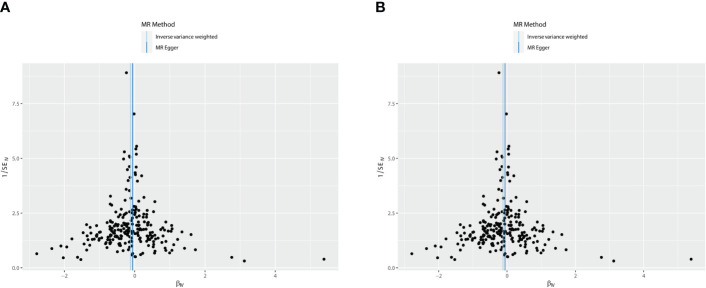
The funnel plot: causal relationship between T2DM and OS. **(A)** Osteoporosis. **(B)** Osteoporosis (UKB-B-17796). T2DM, type 2 diabetes mellitus; OS, osteoporosis.

In the first step, the mean F-value of T2DM instrumental variables was calculated to be 89, indicating that the SNPs used in this study analysis were robust and could avoid potential bias. In the two-sample MR analysis, when OS data from the FinnGen database were used as the outcome variable, the results showed that the incidence of OS decreased by 11% with each 1-SD increase in T2DM risk (*OR* = 0.89, 95% CI = 0.84–0.94, *p* < 0.001), and then the sensitivity analysis of this causal relationship was carried out using the MR-Egger method, and no obvious horizontal pleiotropy was detected (*p*_intercept_ = 0.286). In the analysis of heterogeneity, the consistency analysis was carried out using MR-Egger and IVW methods, and Cochran’s Q statistics showed that there was no heterogeneity (*p*_heterogeneity_ − IVW = 0.716). In the validation dataset, the OS data of “UKB-B-17796” were used, and it was found that contrary to the above results, T2DM was a risk factor for OS (*OR* = 1.05, 95% *CI* = 1.02–1.09, *p* = 0.003), there was no horizontal pleiotropy (*p*_intercept_ = 0.360), and heterogeneity exists (*p*_heterogeneity_ − IVW < 0.001) (**Figures 3**–**5**). For details, see **Tables 1**, **2**, and **Supplementary Table 4**.

**Table 2:** Univariable MR horizontal pleiotropy and heterogeneity.

Horizontal pleiotropy				
Outcome	Egger intercept	Intercept se	*p*_intercept_	
Osteoporosis	−0.005	0.005	0.286	
Osteoporosis (UKB-B-17796)	−0.003	0.003	0.360	
Heterogeneity test				
	Method	Q statistic	Q	*p*_heterogeneity_
Osteoporosis	MR-Egger	220	233	0.719
	Inverse variance weighted	221	234	0.716
Osteoporosis (UKB-B-17796)	MR-Egger	279	178	<0.001
	Inverse variance weighted	281	179	<0.001

MR, Mendelian randomization.

**Figure 6:**
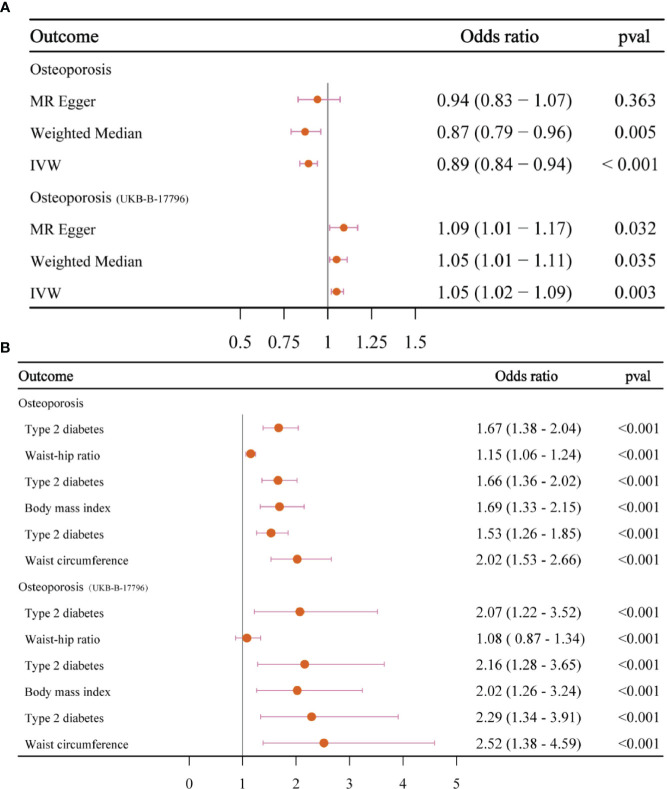
The forest plots. **(A)** The forest plot of the causal relationship between T2DM and OS. **(B)** The forest plot of MVMR results. T2DM, type 2 diabetes mellitus; OS, osteoporosis; MVMR, multivariable Mendelian randomization.

Since the two results were opposite, MVMR analysis was conducted for correction. It was found that T2DM (FinnGen database) became a risk factor; that obesity indicators WHR, BMI, and WC played an important role in the causal relationship between T2DM and OS; and that the *OR* values after correction were 1.67, 1.66, and 1.53, respectively, *p* < 0.05. This result was confirmed in the verification set, and the two maintained a strong causal relationship (**Table 3**; **Supplementary Table 6**; **Figure 6**). In order to exclude the influence of relevant confounders, reverse MR analysis was conducted, and it was found that no relevant SNP was extracted. Subsequently, Steiger’s test was conducted (*p* < 0.05), SNPs related to other traits of T2DM were excluded by the LDtrait tool, and the outcome was consistent with that of MVMR (**Supplementary Tables 5, 7**).

**Table 3:** MVMR estimates for the independent effect of type 2 diabetes on osteoporosis with adjustment for covariates.

Outcome	Method	*OR* (95% *CI*)	*p-*Values
Osteoporosis			
Type 2 diabetes	MV-IVW	1.67 (1.38–2.04)	<0.001
Waist-to-hip ratio		1.15 (1.06–1.24)	0.001
Type 2 diabetes	MV-IVW	1.66 (1.36–2.02)	<0.001
Body mass index		1.69 (1.33–2.15)	<0.001
Type 2 diabetes	MV-IVW	1.53 (1.26–1.85)	<0.001
Waist circumference		2.02 (1.53–2.66)	<0.001
Osteoporosis (UKB-B-17796)			
Type 2 diabetes	MV-IVW	2.07 (1.22–3.52)	0.007
Waist-to-hip ratio		1.08 (0.87–1.34)	0.490
Type 2 diabetes	MV-IVW	2.16 (1.28–3.65)	0.004
Body mass index		2.02 (1.26–3.24)	0.004
Type 2 diabetes	MV-IVW	2.29 (1.34–3.91)	0.003
Waist circumference		2.52 (1.38–4.59)	0.003

MVMR, multivariable Mendelian randomization; IVW, inverse variance weighting.

### Effect of 30 mediating variables on causality between T2DM and OS

In this study, 30 candidate regulatory factors related to glucose metabolism, lipid metabolism, fatty acid metabolism, amino acid metabolism, and inflammatory factors were included, among which 17 regulatory factors strongly related to T2DM were found to be related to glucose metabolism (**Supplementary Table 8**): 1) the FI (*OR* = 1.06, 95% *CI* = 1.04–1.07, *p* < 0.001), 2) FG (*OR* = 1.03, 95% *CI* = 1.02–1.05, *p* < 0.001), 3) 2-H glucose (*OR* = 0.98, 95% *CI* = 0.97–0.99, *p* < 0.001), and 4) HbA1c (*OR* = 1.080, 95% *CI* = 1.06–1.10, *p* < 0.001). Lipid metabolism index was related to 1) Apo A-I (*OR* = 0.99, 95% *CI* = 0.98–0.99, *p* = 0.011), 2) HDL-C (*OR* = 0.980, 95% *CI* = 0.97–0.99, *p* = 0.001), 3) the LDL-C (*OR* = 1.016, 95% *CI* = 1.01–1.03, *p* = 0.001), and 4) TG (*OR* = 1.04, 95% *CI* = 1.02–1.06, *p* < 0.001). Fatty acid metabolism was related to 1) SFA (*OR* = 1.016, 95% *CI* = 1.00–1.03, *p* = 0.018) and 2) MUFA (*OR* = 1.03, 95% *CI* = 1.01–1.04, *p* = 0.001). Amino acid was correlated to 1) isoleucine (*OR* = 1.04, 95% *CI* = 1.03–1.06, *p* < 0.001), 2) leucine (*OR* = 1.05, 95% *CI* = 1.03–1.06, *p* < 0.001), 3) valine (*OR* = 1.05, 95% *CI* = 1.04–1.07, *p* < 0.001), 4) phenylalanine (*OR* = 1.03, 95% *CI* = 1.01–1.04, *p* < 0.001), 5) tyrosine (*OR* = 1.03, 95% *CI* = 1.01–1.05, *p* < 0.001), 6) alanine (*OR* = 1.05, 95% *CI* = 1.03–1.07, *p* < 0.001), and 7) histidine (*OR* = 1.03, 95% *CI* = 1.01–1.01, *p* < 0.001).

We used the MR-Egger method to detect the horizontal pleiotropy of 17 mediating factors, among which tyrosine showed horizontal pleiotropy (*p*_intercept_ = 0.286). There was heterogeneity in Cochran’s Q test (*p* < 0.001) (**Supplementary Tables 9, 10**). Then, tyrosine data from http://www.phenoscanner.medschl.cam.ac.uk/ ruled out confounding SNPs, horizontal pleiotropic, and heterogeneity.

Finally, the MVMR analysis of T2DM and 17 candidate mediating factors with OS showed no causal association between T2DM and OS (**Supplementary Table 11**), and no evidence proved that mediating variables played a role in the causal association between T2DM and OS.

## Discussion

According to the results of this study, there is a strong causal association between T2DM and OS. With the aggravation of diabetes, the risk of OS increases, and the pathogenesis is closely related to obesity. To our knowledge, this study is the first to include glucose metabolism, lipid metabolism, amino acid metabolism, and inflammatory factors in the causal analysis of T2DM and OS.

T2DM has a high genetic predisposition and is often associated with unhealthy diet and obesity. In a randomized controlled study of 73,105 patients with abdominal obesity, most of whom had diabetes, most of these patients had T2DM, and the results showed that abdominal fat thickness increased the incidence of OS by 23% (34). With the deepening understanding of bone, researchers have found that bone is not only a weight-bearing organ but also an endocrine organ. It can secrete osteocalcin (OCN), lipocalin-2 (LCN2), sclerostin (SOST), and fibroblast growth factor-23 (FGF-23) to regulate patients’ appetite and blood sugar, and it can be regulated by other endocrine organs (35–37). Many fundamental studies have proved that T2DM has a damaging effect on bone. Physiological insulin doses can promote osteoblast proliferation and collagen synthesis and inhibit osteoclast activity. T2DM patients are often accompanied by insulin deficiency, decreased bone density, and bone trabecular density, resulting in further aggravation of OS. Similar to previous studies, after excluding the possible effects of obesity-related indicators, we found that T2DM still maintained a robust causal relationship with OS, and following the possession of T2DM, which can aggravate the severity of OS, different severity of T2DM may cause different damage to OS.

This study screened 30 traits related to metabolism and inflammation to explore the possible causal relationship between metabolic factors. Seventeen metabolic traits were found to have a causal relationship with T2DM and were subsequently associated with OS to explore whether they mediate the regulatory effect. However, no metabolic factors play a role in the causal relationship. Given this result, the disorders of glucose metabolism, lipid metabolism, amino acid metabolism, and inflammatory metabolism are mostly related to acquired life habits. In the early stage of T2DM patients, bone mineral density not only does not decrease but increases (38). With the aggravation of diabetes, advanced glycation end products (AGEs) will accumulate in bone cells and then cause mitochondrial swelling (39), induce iron death, and produce a large number of reactive oxygen species, resulting in a decrease in the number of osteoblasts and aggravation of OS (8). Circulating amino acids are related to OS and the fracture risk caused by it. After the MR analysis of 5,724 fracture cases, it was found that valine was strongly related to hip fracture, and the incidence rate increased by 0.79% when the blood valine content increased by 1-SD (40). In recent years, the musculoskeletal system has gradually been regarded as a whole, and the two are close in space and affect each other in physiological function. Valine, leucine, and isoleucine are essential for skeletal muscle metabolism (41). In addition, tryptophan also plays a pivotal role. With the increasing age and the progression of T2DM, the intermediate metabolite of tryptophan, Kyn, can also cause neurodegeneration, acrodynia, and OS (42). Inflammatory factors play a role in the pathogenesis of OS. It is well known that T2DM can cause inflammatory damage to the liver and pancreas and subacute inflammation through RANKL (an effective NF-κB stimulator), leading to β-cell failure. When denosumab inhibits “RANKL”, subacute inflammation and insulin resistance can be improved (43–45). However, this study did not find that inflammatory factors mediate the causal relationship between T2DM and OS, but this result still needs to be considered. Because inflammatory factors are often produced under the stimulation of pathological environment, which can cause abnormal signaling of osteogenic pathway and further cause bone damage, more studies are needed to explore the relationship between inflammatory factors and osteogenic pathway factors to clarify the pathogenic mechanism of inflammatory factors.

When we initially explored the causal relationship between T2DM and OS, T2DM was a protective factor for OS. After analyzing obesity-related indicators, T2DM was translated into a risk factor, which was validated in the MRC-IEU database. We believe that the reasons for this result are as follows. 1) According to the basic experimental results, the damage caused by T2DM to the human body includes not only sugar toxicity but also lipid toxicity. In the previous study of our research group, it was found that the damage caused by high fat was higher than that in the high-sugar environment. *In vitro*, palmitic acid (PA) was used to simulate the high-fat environment, and the concentration of PA below 0.2 mmol/L had no obvious effect on osteoblasts and even played a promoting role. When the concentration was higher than 0.25 mmol/L, the osteoblast apoptosis would occur sharply, and the expression of osteoblast protein was significantly reduced. In animal experiments, we divided mice into a control group, a high-fat diet group, and a high-fat diet combined with the T2DM modeling group and found that bone mineral density increased in the high-fat diet group, but bone fragility also increased. The high-fat diet combined with the T2DM modeling group showed obvious bone destruction, decreased bone density, and serious OS, so we believe that this is the reason for the different results before excluding obesity-related traits. We then compared the two databases. The FinnGen database had a sample size of 212,778, which is much smaller than that of the MRC-IEU database (n = 463,010). Although we chose a database with a large sample size and a long recent release time whenever possible, it is easy to lead to bias compared to the MRC-IEU database due to insufficient sample size. 2) In the process of data analysis, we could not obtain the original data of OS (FinnGen database), and it is not clear whether the original data were stratified for different obesity degrees and ages, so the role of obesity-related indicators should be clarified.

The advantages of this paper are as follows. 1) Based on the previous basic research and sufficient MR analysis, the effects of obesity, various metabolic substances, and inflammatory substances are considered. 2) Based on the inclusion of an extensive sample database, the F-value of the IVs selected is far greater than 10, and complementary sensitivity analysis methods are adopted to improve the robustness of MR results. 3) The MRC-IEU database is used as the verification set of OS to improve statistical efficiency and accuracy. However, this study still has limitations. 1) There are many factors affecting diabetes, and the manifestations are different in different disease periods. The different traits of T2DM are currently lacking, so the results cannot be accurately discussed. 2) The patients in the OS databases were from a European population, and the original text could not be found, so the correction factors in the articles could not be analyzed to ensure that the baseline data of the population were similar, which limited the promotion of the results in different races, genders, and ages. 3) Diabetes is a common but complex disease, and the combination of blood sugar and blood lipid at different stages of onset will have different effects on OS; OS includes bone density, trabecular separation, bone fragility, cortical bone thickness, and other indicators and requires strict stratification to better promote MR analysis to produce more accurate results. We look forward to publishing a database of different blood glucose levels, lipid levels, and obesity stratification in the future, which may yield different results.

In conclusion, our research confirms a robust causal relationship between T2DM and OS and uncovers the role of obesity as a mediator in this relationship. These findings significantly contribute to understanding the T2DM-OS risk connection, providing a solid foundation for future studies in this field. We eagerly anticipate the publication of larger sample sizes and more diverse ethnic data, enabling us to conduct even more precise analyses of T2DM and OS.

## Conclusion

This study found that T2DM is a negative factor for OS, there is a robust causal relationship between them, and obesity-related traits play an essential role. Our study showed that the causal relationship between T2DM and OS was not directly related to glucose metabolism, lipid metabolism, fatty acid metabolism, amino acid metabolism, and inflammatory factors. Our study reveals the genetic pathogenesis of OS and provides new ideas for treating patients with OS combined with metabolic disorders in the future.

## Data availability statement

The datasets presented in this study can be found in online repositories. The names of the repository/repositories and accession number(s) can be found in the article/**Supplementary Material**.

## Author contributions

QY: Conceptualization, Data curation, Writing – original draft. XW: Writing – review & editing, Investigation. YL: Writing – review & editing, Supervision. JL: Writing – original draft, Data curation. DZ: Writing – original draft, Writing – review & editing, Supervision.
